# Unmasking the Hidden Struggle Behind the White Coat: Screening Adult ADHD Symptoms Among Medical Students at the University of Tabuk, Saudi Arabia (2025)

**DOI:** 10.3390/healthcare13131528

**Published:** 2025-06-26

**Authors:** Zinab Alatawi

**Affiliations:** Department of Family and Community Medicine, Faculty of Medicine, University of Tabuk, Tabuk 71491, Saudi Arabia; zalatawi@ut.edu.sa

**Keywords:** ADHD, adult attention-deficit/hyperactivity disorder, medical students, Saudi Arabia, ASRS-v1.1, prevalence, mental health, academic stress

## Abstract

Background: Attention-deficit/hyperactivity disorder (ADHD) is a chronic neurodevelopmental disorder that continues into adulthood and is linked to significant academic, occupational, and psychological challenges. Medical students may be at heightened risk due to the cognitive and emotional demands of their training. However, regional data on adult ADHD symptoms in this population, particularly in northern Saudi Arabia, remain limited. Objective: to estimate the prevalence of probable adult ADHD symptoms among medical students at the University of Tabuk and to examine the associated sociodemographic, academic, and health-related factors. Methods: A cross-sectional, web-based survey was conducted between 15 May and 5 June 2025 among randomly selected Saudi medical students (years 2–6) at the University of Tabuk. The validated Arabic version of the WHO Adult ADHD Self-Report Scale (ASRS-v1.1) was used to screen for probable ADHD. A positive screen was defined as ≥4 flagged items. Descriptive and bivariate analyses were performed using SPSS v29. Results: Of the 219 respondents (60.3% male; mean age: 21.6 years), 23.3% screened positive for probable adult ADHD. Symptom frequency peaked in the third (40.0%) and fourth (35.6%) academic years and was lowest among sixth-year students (11.4%) (*p* = 0.012). A strong association was observed between positive ADHD screening and self-reported psychiatric disorders (*p* < 0.001). No statistically significant associations were found for gender, income, GPA, marital status, or academic phase. Conclusions: Nearly one in four medical students at the University of Tabuk exhibited symptoms suggestive of adult ADHD, a prevalence markedly higher than global estimates and consistent with regional trends. The association with psychiatric morbidity and the mid-programme peak suggests a need for proactive screening, mental health support, and academic accommodations. Universities can translate these findings into practice by instituting routine ADHD screening, offering flexible assessment accommodations, embedding peer-mentoring programmes, and strengthening on-campus mental-health referral pathways.

## 1. Introduction

Attention-deficit/hyperactivity disorder (ADHD) is a chronic neurodevelopmental condition characterised by persistent patterns of inattention, hyperactivity, and impulsivity that often persist into adulthood [1,2]. While the overt manifestations of ADHD may evolve with age, a significant proportion of affected individuals continue to experience substantial functional impairments in academic, occupational, and interpersonal domains [3]. A recent umbrella review of five systematic reviews estimated the pooled global prevalence of adult ADHD at approximately 3.1%, highlighting its relevance as a public health concern [4].

In university settings, these impairments manifest as academic underperformance, reduced grade point averages, increased dropout rates and heightened psychological distress, and they also expose affected students to intense psychosocial stressors—including peer bullying, social isolation, and feelings of alienation—that further amplify burnout risk and programme attrition [5]. In Saudi Arabia, the burden appears to be even greater. A 2023 systematic review and meta-analysis reported a pooled adult ADHD prevalence of 12.4%, considerably higher than the global average [6]. However, data focusing specifically on adult ADHD in university students—particularly those enrolled in rigorous programmes such as medicine—remain scarce and regionally inconsistent.

Medical students may be especially vulnerable because of the demanding cognitive, emotional, and time commitments of their training [7]. Previous cross-sectional studies from different regions of Saudi Arabia have reported a wide range of ADHD symptom prevalence among medical students, from 10.9% in Riyadh [8] and 11.9% in Jeddah [9] to 26% in the Eastern Province [10], suggesting substantial heterogeneity possibly influenced by institutional, environmental, or methodological differences. These rates not only exceed estimates from the general population but also underscore the potential for undetected cognitive and psychological difficulties in this academically at-risk group.

Moreover, positive ADHD screening in medical students has been associated with increased levels of anxiety, depressive symptoms, and excessive screen time [11]. Despite these findings, awareness and understanding of ADHD remain limited among medical trainees; for example, only one-third of students at Qassim University demonstrated adequate knowledge of the disorder [12]. This lack of awareness, combined with high levels of stress and stigma, may contribute to underdiagnosis and inadequate support for students experiencing symptoms.

The World Health Organization’s six-item Adult ADHD Self-Report Scale (ASRS-v1.1) is a brief, validated, and widely used screening instrument designed to identify adults at risk for ADHD in general and clinical populations [13]. To date, no published studies have explored the prevalence and correlates of ADHD symptoms among medical students in the northern region of Saudi Arabia. The present study, therefore, aimed to estimate the prevalence of probable adult ADHD symptoms among medical students at the University of Tabuk and to examine their association with key demographic, academic, and mental health factors. Addressing this evidence gap, the study seeks to inform targeted interventions and support strategies for at-risk students in medical education settings.

## 2. Methods

### 2.1. Study Design and Setting

This study employed a cross-sectional, web-based survey design to assess the prevalence of adult ADHD symptoms among medical students. Data collection took place from 15 May to 5 June 2025, targeting students enrolled at the College of Medicine, University of Tabuk, located in northwestern Saudi Arabia.

### 2.2. Participants

Eligible participants included Saudi nationals of both sexes, currently enrolled in academic years two through six. Inclusion was contingent upon providing electronic informed consent. Preparatory-year students, non-Saudi nationals, and those who declined to participate were excluded from the study.

### 2.3. Sample Size and Sampling Strategy

To ensure an adequate sample size, the single-proportion formula was applied, assuming a conservative ADHD prevalence of 50%, with a 95% confidence level and a margin of error of 5%. This yielded a minimum required sample of 211 students. A comprehensive list of eligible students was obtained from the college administration and served as the sampling frame. A simple random sampling technique was implemented using computer-generated random numbers, and selected participants were invited to participate via their institutional e-mail accounts. Informed consent was signed electronically before participation in a Google form. To check for selection bias, we compared the gender and academic year distributions of respondents and non-respondents using a Chi-square test; no significant differences were found.

### 2.4. Data Collection Instrument

Data were collected using the validated Arabic version of the World Health Organization’s six-item Adult ADHD Self-Report Scale (ASRS-v1.1) [13]. Each item is rated on a five-point Likert scale ranging from “never” (0) to “very often” (4). A positive screen for probable adult ADHD was defined according to WHO guidelines: four or more items marked as flagged items that are either “sometimes” (score of 2), “often” (score of 3), or “very often” (score of 4) for the first three items, and “often” (score of 3) or “very often” (score of 4) for the last three items [14]. A pilot study in 30 students not included in the analysis yielded a Cronbach’s alpha value of 0.75, indicating acceptable reliability.

In addition to ADHD symptoms, the questionnaire gathered sociodemographic and academic data, including age, sex, marital status, family income, academic year, grade point average (GPA), and clinical phase of training (pre-clinical refers to those in the second and third years, while clinical refers to those in the last three years of medical school), as well as self-reported history of chronic medical or psychiatric conditions.

### 2.5. Data Collection Procedure

All participants received a study invitation e-mail containing a brief description of the research objectives, assurances of confidentiality, and the voluntary nature of participation. The first page of the electronic survey included a digital informed consent form; only students who confirmed consent were able to proceed to the questionnaire. The survey remained open for a duration of four weeks, with weekly e-mail reminders sent to non-respondents.

### 2.6. Study Variables

The primary outcome variable was a positive screen for probable adult ADHD based on the ASRS scoring algorithm. Independent variables included demographic (age, sex, marital status, income), academic (GPA, year of study, clinical phase), and health-related (chronic illness, psychiatric history) characteristics.

### 2.7. Data Management and Statistical Analysis

Upon survey closure, responses were exported from Google Forms to Microsoft Excel, cleaned, and imported into IBM SPSS Statistics Version 29 for analysis. Descriptive statistics were used to summarise the distribution of participant characteristics. The prevalence of probable ADHD was estimated with corresponding 95% confidence intervals.

Bivariate associations between ADHD screening status and independent variables were assessed using Pearson’s Chi-square test or Fisher’s exact test, as appropriate, for categorical variables. A two-sided *p*-value of less than 0.05 was considered indicative of statistical significance.

The data collection tool was designed to require all fields to be completed; therefore, there was no missing data.

### 2.8. Ethical Considerations

Prior to data collection, the study protocol received ethical approval from the University of Tabuk Local Research Ethics Committee (Approval ID: HAP-07-TU-001). Participation in the study was entirely anonymous, with no personally identifiable information collected. All data were securely stored on a password-protected computer accessible only to the research team. The study was conducted in accordance with the principles of the Declaration of Helsinki and adhered to all ethical regulations of the University of Tabuk.

## 3. Results

### 3.1. Sociodemographic, Academic and Health Characteristics

The study enrolled 219 medical students (response rate 103%) whose median profile was male, single, and financially constrained. Slightly more than half were aged 21–23 years (51.1%), while nearly one-third were younger than 20 years. Men predominated (60.3%), yielding a male-to-female ratio of 1.5:1. Most respondents reported a monthly family income below 5000 SAR (88.1%) and had not yet married (97.7%). Although first admitted only two years earlier, many participants had already achieved strong academic standing: one-third were in the second academic year, yet 51.6% recorded a GPA ≥ 4.5 on a 5-point scale. Students were split between the pre-clinical (44.3%) and clinical (55.7%) training phases. Chronic somatic illness (9.1%) and self-declared psychiatric disorders (6.4%) were relatively uncommon (Table 1).

### 3.2. Frequency of ADHD-Related Symptoms and Screening Outcome

**Figure 1:**
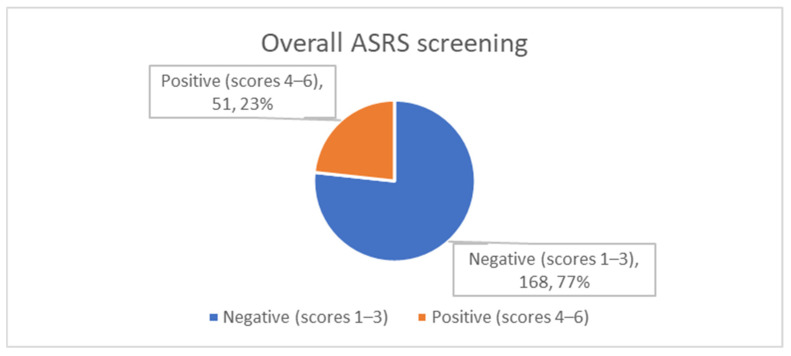
Overall ASRS screening status (N = 219).

Responses to the six-item ASRS-v1.1 revealed that sub-threshold ADHD manifestations were widespread. “Sometimes” was the modal reply for four items—trouble finishing details, difficulty organising tasks, forgetfulness, and feeling “driven by a motor”—suggesting intermittent inattentive and hyperactive behaviours across the cohort. Notably, one in five students (21.9%) reported fidgeting or squirming “very often”, and 16.4% frequently avoided cognitively demanding tasks. When the ASRS-v1.1 scoring algorithm was applied, 23.3% of students screened positive for probable adult ADHD (Table 2, Figure 1).

### 3.3. Factors Associated with a Positive ADHD Screen

Bivariate Chi-square (χ^2^) analyses identified two background characteristics that significantly correlated with a positive screen. First, ADHD positivity varied by academic year (*p* = 0.012): it peaked in the third year (40.0%) and fourth year (35.6%), then declined markedly by the sixth year (11.4%). Second—and most strikingly—students who self-reported a psychiatric disorder were roughly four times more likely to screen positive than their peers without such a history (78.6% vs. 19.5%; *p* < 0.001). Associations with age, gender, monthly income, marital status, GPA tier, academic phase, and chronic physical illness did not reach statistical significance, although a non-significant trend suggested higher ADHD positivity among students reporting a chronic health condition (40.0% vs. 21.6%) (Table 3).

## 4. Discussion

This study provides novel insight into the burden of adult attention-deficit/hyperactivity disorder (ADHD) symptoms among medical students in northern Saudi Arabia, a population in which such data have been scarce. Using the WHO Adult ADHD Self-Report Scale (ASRS-v1.1), we found that 23.3% of students screened positive for probable ADHD, a prevalence markedly higher than global estimates. The findings demonstrate a pronounced clustering of symptoms in the middle years of medical training and reveal a significant association with self-reported psychiatric morbidity, highlighting the intersection of academic stress, mental health vulnerability, and neurodevelopmental symptomatology in this context.

When situated within the national literature, our prevalence surpasses those reported in Riyadh (10.9%) [8] and Jeddah (11.9%) [9], and is higher than rates observed in Hofuf (14%) [15], but remains lower than the 26% found in the Eastern Province [10]. All of these values exceed the general Saudi adult prevalence of 12.4% estimated in recent meta-analyses [6], suggesting that medical students are a high-risk subgroup.

Comparatively, the prevalence in our cohort is lower than that observed in first-year university students in the United Arab Emirates (34.7%) [16], and similar to figures from Pakistan (34.8%) [17], yet notably higher than estimates from Iran (6–8%) [18] and China (3–4%) [19]. These regional differences may reflect variations in academic systems, sociocultural attitudes toward mental health, and the application of different screening tools and diagnostic thresholds. Globally, the prevalence of symptomatic adult ADHD is estimated to be around 6–7% in some reviews and 3.1% in an umbrella review; our observed rate was nearly four to six times the international average [4,20].

A key pattern observed in our data was the elevated prevalence of ADHD symptoms in students in their third and fourth academic years, which declined notably in the sixth year. This trend aligns with findings from the Eastern Province [10] and King Faisal University [15], where early academic stages appeared to be associated with increased ADHD positivity. Conversely, a study from Riyadh reported no significant variation across academic years [8], indicating that institutional and curricular factors may modulate the emergence or detection of symptoms. No significant sex-based differences were observed in our cohort, corroborating results from Riyadh and Jeddah [8,9], though contrasting with findings from the UAE and Kenya, where women were more frequently affected [16,21].

While students with lower GPAs exhibited a non-significant trend toward increased ADHD symptomatology, a definitive association with academic performance could not be established in our sample. This diverges from evidence in Jeddah, where poor academic achievement independently predicted ADHD positivity [9], yet aligns with results from the Eastern Province showing no significant correlation [10]. Furthermore, socio-economic indicators such as family income and marital status were not associated with ADHD in this study, mirroring patterns reported in Riyadh but differing from Jeddah and Eastern Province data, where high income and unmarried status, respectively, were linked to increased risk [9,10].

The most prominent association emerged between ADHD symptoms and self-reported psychiatric morbidity. Students with a history of psychiatric disorders were significantly more likely to screen positive, echoing similar findings from studies in the Eastern Province [10], Jeddah [9], and Pakistan [17], as well as international data from China linking ADHD with heightened risks of anxiety, depression, and suicidal ideation [19]. This reinforces the clinical imperative to address co-occurring mental health concerns in students exhibiting ADHD traits, as such comorbidities may compound functional impairments and reduce help-seeking behaviour.

From a practical standpoint, medical schools can adopt evidence-based measures to maximise the engagement and success of students who screen positive for ADHD. First, assessment policies can be diversified by offering flexible examination formats—such as oral viva voce, take-home, or computer-based tests with extended time windows—that emphasise applied clinical reasoning rather than rapid factual recall. Second, periodic, individualised performance reviews can provide formative feedback and allow early remediation should difficulties emerge. Third, structured peer-support (‘buddy’) and faculty-mentoring programmes have consistently been shown to reduce isolation, improve time-management skills, and foster professional identity formation among neurodivergent trainees. Finally, campus-wide anti-stigma campaigns that communicate ADHD as a neurodevelopmental difference—not a deficit—can cultivate an inclusive learning climate [7].

This study benefits from the use of a validated, internationally recognised screening tool (ASRS-v1.1), the complete enumeration of a defined medical student population, and detailed stratification across academic years. Nevertheless, several limitations must be acknowledged. The single-institution, cross-sectional design limits generalisability. The reliance on self-reported data may introduce recall and reporting biases, and the absence of clinical diagnostic interviews may result in overestimation of prevalence due to symptom overlap with other conditions. Consequently, our prevalence estimate should be interpreted as the proportion of students at high risk for ADHD rather than the definitive disorder prevalence. Because no power analysis was done for the association tests, some non-significant results could simply reflect an inadequate sample size—especially for smaller effect sizes—rather than a real lack of association. Finally, given ADHD’s neurodevelopmental origins, our cross-sectional design cannot establish causality, and the elevated screening rate may reflect either the selection of neurodivergent students into medicine or the way medical school stressors accentuate latent symptoms, or both.

## 5. Conclusions

Almost one in four medical students at the University of Tabuk screened positive for probable adult ADHD, a figure substantially higher than global estimates and comparable to regional cohorts. Symptom burden peaked in the middle academic years and was strongly intertwined with self-reported psychiatric morbidity, underscoring a critical period when academic and psychological pressures converge. These findings highlight the need for routine ADHD screening, confidential mental health services, and targeted academic accommodations within Saudi medical colleges. Multi-centre studies using structured diagnostic interviews are warranted to refine national prevalence estimates and to unpack curricular or environmental stressors contributing to symptom expression. Early, integrated interventions could mitigate the academic underperformance and psychological distress associated with undetected ADHD, ultimately supporting student well-being and professional development.

## Figures and Tables

**Table 1:** Sociodemographic, academic, and health profiles of medical students, University of Tabuk (N = 219).

Variable	Category	n	%
Age (years)	<20	63	28.8
	21–23	112	51.1
	24–26	44	20.1
Gender	Female	87	39.7
	Male	132	60.3
Monthly family income	<5000 SAR	193	88.1
	5000–10,000 SAR	10	4.6
	10,000–20,000 SAR	8	3.7
	>20,000 SAR	8	3.7
Marital status	Single	214	97.7
	Married	4	1.8
	Divorced/widow	1	0.5
Academic year	2	71	32.4
	3	25	11.4
	4	45	20.5
	5	34	15.5
	6	44	20.1
GPA	<3.0	9	4.1
	3.0–3.49	15	6.8
	3.5–3.99	29	13.2
	4.0–4.49	53	24.2
	4.5–5.0	113	51.6
Academic phase	Pre-clinical	97	44.3
	Clinical	122	55.7
Chronic health disease	Yes	20	9.1
	No	199	90.9
Psychiatric disorder	Yes	14	6.4
	No	205	93.6

**Table 2:** Distribution of responses to the six-item ASRS-v1.1 (N = 219).

Item	Never n (%)	Rarely n (%)	Sometimes n (%)	Often n (%)	Very Often n (%)
1. How often do you have trouble wrapping up the final details of a project, once the challenging parts have been done?	33 (15.1)	66 (30.1)	82 (37.4)	29 (13.2)	9 (4.1)
2. How often do you have difficulty getting things in order when you have to do a task that requires organization?	37 (16.9)	75 (34.2)	62 (28.3)	31 (14.2)	14 (6.4)
3. How often do you have problems remembering appointments or obligations?	58 (26.5)	75 (34.2)	60 (27.4)	19 (8.7)	7 (3.2)
4. When you have a task that requires a lot of thought, how often do you avoid or delay getting started?	28 (12.8)	43 (19.6)	70 (32.0)	42 (19.2)	36 (16.4)
5. How often do you fidget or squirm with your hands or feet when you have to sit down for a long time?	31 (14.2)	38 (17.4)	50 (22.8)	52 (23.7)	48 (21.9)
6. How often do you feel overly active and compelled to do things, like you were driven by a motor?	31 (14.2)	61 (27.9)	88 (40.2)	23 (10.5)	16 (7.3)

**Table 3:** Crosstabulation of factors associated with positive ADHD screening (ASRS-v1.1, N = 219).

Variable	Category	Negative n (%)	Positive n (%)	Total n	Χ^2^ *p*-Value
Age (years)	<20	51 (81.0)	12 (19.0)	63	0.155
	21–23	80 (71.4)	32 (28.6)	112	
	24–26	37 (84.1)	7 (15.9)	44	
Gender	Female	66 (75.9)	21 (24.1)	87	0.809
	Male	102 (77.3)	30 (22.7)	132	
Monthly income (SAR)	<5000	144 (74.6)	49 (25.4)	193	0.145
	5000–10,000	8 (80.0)	2 (20.0)	10	
	10,000–20,000	8 (100)	0 (0.0)	8	
	>20,000	8 (100)	0 (0.0)	8	
Marital status	Single	164 (76.6)	50 (23.4)	214	0.105
	Married	4 (100)	0 (0.0)	4	
	Divorced/Widow	0 (0.0)	1 (100)	1	
Academic year	2nd	59 (83.1)	12 (16.9)	71	0.012 *
	3rd	15 (60.0)	10 (40.0)	25	
	4th	29 (64.4)	16 (35.6)	45	
	5th	26 (76.5)	8 (23.5)	34	
	6th	39 (88.6)	5 (11.4)	44	
GPA	<3.0	7 (77.8)	2 (22.2)	9	0.106
	3.0–3.49	12 (80.0)	3 (20.0)	15	
	3.5–3.99	18 (62.1)	11 (37.9)	29	
	4.0–4.49	37 (69.8)	16 (30.2)	53	
	4.5–5.0	94 (83.2)	19 (16.8)	113	
Academic phase	Pre-clinical	75 (77.3)	22 (22.7)	97	0.850
	Clinical	93 (76.2)	29 (23.8)	122	
Chronic health disease	No	156 (78.4)	43 (21.6)	199	0.064
	Yes	12 (60.0)	8 (40.0)	20	
Psychiatric disorder	No	165 (80.5)	40 (19.5)	205	<0.001 *
	Yes	3 (21.4)	11 (78.6)	14	

* Statistically significant at α = 0.05.

## Data Availability

The data presented in this study are available upon request from the corresponding author.

## References

[1] Magnus W., Anilkumar A.C., Shaban K. Attention Deficit Hyperactivity Disorder. StatPearls, August 2023. https://www.ncbi.nlm.nih.gov/books/NBK441838/.

[2] American Psychiatric Association (2022). Diagnostic and Statistical Manual of Mental Disorders.

[3] Abdelnour E., Jansen M.O., Gold J.A. (2022). ADHD Diagnostic Trends: Increased Recognition or Overdiagnosis?. Mo. Med..

[4] Ayano G., Tsegay L., Gizachew Y., Necho M., Yohannes K., Abraha M., Demelash S., Anbesaw T., Alati R. (2023). Prevalence of attention deficit hyperactivity disorder in adults: Umbrella review of evidence generated across the globe. Psychiatry Res..

[5] DuPaul G.J., Weyandt L.L., O’Dell S.M., Varejao M. (2009). College students with ADHD: Current status and future directions. J. Atten. Disord..

[6] Aljadani A.H., Alshammari T.S., Sadaqir R.I., Alrashede N.O.E., Aldajani B.M., Almehmadi S.A., Altuhayni A.S., Abouzed M.A. (2023). Prevalence and Risk Factors of Attention Deficit-Hyperactivity Disorder in the Saudi Population: A Systematic Review and Meta-analysis. Saudi J. Med. Med. Sci..

[7] Godfrey-Harris M., Shaw S.C.K. (2023). The experiences of medical students with ADHD: A phenomenological study. PLoS ONE.

[8] Alrahili N., Aldakheel A., AlUbied A., Almalki A., AlBarrak A., Al-Dosari B., Alhemaidi W., Alageel A. (2019). Prevalence of Adult Attention Deficit Hyperactivity Disorder among medical students in Riyadh City. Int. J. Med. Dev. Ctries..

[9] Alghamdi W.A., Alzaben F.N., Alhashemi H.H., Shaaban S.S., Fairaq K.M., Alsuliamani A.S., Mahin B.A., Ghurab R.A., Sehlo M.G., Koenig H.G. (2022). Prevalence and correlates of attention deficit hyperactivity disorder among college students in Jeddah, Saudi Arabia. Saudi J. Med. Med. Sci..

[10] Alsafar F.A., Alsaad A.J., Albukhaytan W.A. (2024). Prevalence of adult attention deficit hyperactivity disorder (ADHD) among medical students in the Eastern Province of Saudi Arabia. Saudi Med. J..

[11] Ferreira J.S.N., Da Silva R.M., Hamuche C.F., Nascimento R.B.D., Ribeiro A.P., Gil S., Neves L.M. (2025). Positive ADHD Scores are Associated with Higher Screen Time and Anxiety Symptoms in Medical Students: Cross-sectional Study. Actas Esp. Psiquiatr..

[12] Alsuhaibani M., Alsaawi O., Alsuwayti K., Alahmed I. (2020). Awareness and knowledge of attention deficit and hyperactivity disorder among medical students of Qassim University in Saudi Arabia. J. Fam. Med. Prim. Care.

[13] Kessler R.C., Adler L., Ames M., Demler O., Faraone S., Hiripi E., Howes M.J., Jin R., Secnik K., Spencer T. (2005). The World Health Organization adult ADHD self-report scale (ASRS): A short screening scale for use in the general population. Psychol. Med..

[14] World Health Organization (WHO) (2005). Adult ADHD Self-Report Scale (ASRS-v1.1) Symptom Checklist Instructions.

[15] Begum N., Pathath A.W., Elballah K., Almubireek M.A., Irshad S., Ali S.I. (2024). Prevalence of Adult Attention Deficit Hyperactivity Disorder among Medical Students, King Faisal University. South East. Eur. J. Public Health.

[16] Al-Yateem N., Slewa-Younan S., Halimi A., Saeed S.A., Tliti D., Mohammad M., Ridwan M., Zeidan R., Hammash M.H., Ahmed F.R. (2024). Prevalence of Undiagnosed Attention Deficit Hyperactivity Disorder (ADHD) Symptoms in the Young Adult Population of the United Arab Emirates: A National Cross-Sectional Study. J. Epidemiol. Glob. Health.

[17] Sabir H., Khan M., Imran K., Nisa Z.U., Amer S.A. (2024). The prevalence of undiagnosed attention-deficit/hyperactivity disorder among undergraduate medical students: A survey from Pakistan. BMC Psychiatry.

[18] Mosalanejad M., Mosalanejad L., Lashkarpour K. (2013). Prevalence of ADHD Among Students of Zahedan University of Medical Science in Iran. Iran J. Psychiatry Behav. Sci..

[19] Shen Y., Chan B.S.M., Liu J., Meng F., Yang T., He Y., Lu J., Luo X., Zhang X.Y. (2018). Estimated prevalence and associated risk factors of attention deficit hyperactivity disorder (ADHD) among medical college students in a Chinese population. J. Affect. Disord..

[20] Song P., Zha M., Yang Q., Zhang Y., Li X., Rudan I. (2021). The prevalence of adult attention-deficit hyperactivity disorder: A global systematic review and meta-analysis. J. Glob. Health.

[21] Atwoli L., Owiti P., Manguro G., Ndambuki D. (2011). Attention deficit hyperactivity disorder symptom self-report among medical students in Eldoret, Kenya. Afr. J. Psychiatry.

